# Where are the RNA vaccines for TB?

**DOI:** 10.1080/22221751.2021.1935328

**Published:** 2021-06-18

**Authors:** Xiao-Yong Fan, Douglas Bruce Lowrie

**Affiliations:** TB Center, Shanghai Emerging and Re-emerging Institute, Shanghai Public Health Clinical Center and Key Laboratory of Medical Molecular Virology of MOE/MOH, Fudan University, Shanghai, People’s Republic of China

**Keywords:** Tuberculosis, RNA vaccination, vaccine, in vitro transcription, COVID-19

## Abstract

A simple mRNA vaccine was shown to protect mice against tuberculosis more than 15 years ago. Like COVID-19, tuberculosis is a respiratory infection killing over a million people per year. It too presents a global emergency. Can the stunning success of RNA vaccination against COVID-19 be replicated for TB?

The speed of development, deployment and high efficacy of the new RNA vaccines against COVID-19 is stunning!

The emerging disease was rapidly recognized as presenting a global health crisis and provided the necessary incentives for this major breakthrough for vaccinology [1]. Early projections from available data suggested that the disease might kill millions of people worldwide. Sadly, the projections have proven appropriate, but the RNA vaccines will play a major part in countering this horrific disease [2,3].

Tuberculosis, like COVID-19, will also kill over a million people this year and, notably, it does so every year [4], despite being declared a “Global Emergency” by WHO in 1993 [5]. Efforts to prevent this are being compromised by the lack of an adequate vaccine and the emergence and spread of multi-drug-resistant TB (MDR-TB) bacteria [4]. Could the development and deployment of RNA vaccines against TB have an efficacy comparable to that against COVID-19?

The demonstration in 2004 of a protective effect of RNA vaccination against tuberculosis in mice was one of the first proofs of concept for RNA vaccines [6]. In that study, messenger RNA (mRNA) was transcribed from DNA encoding an antigen of *Mycobacterium tuberculosis*, purified, and the naked *in-vitro*-transcribed (ivt) mRNA was injected four times into the skin of the mice at 3-week intervals. When challenged with virulent *M. tuberculosis* infection four weeks later the mice were significantly protected. The protection was less than that obtained with BCG, the only anti-TB vaccine that is currently available for human use. The finding has not been followed up. Perhaps on the basis that development of practical ivt RNA vaccines would be too difficult and expensive, attention shifted to DNA vaccines.

The subsequent research, development and application of RNA (and DNA) vaccines in general has tended to focus upon potentially lucrative targets such as therapies for allergies and cancers. Nevertheless, some progress has been made towards protective RNA vaccines against some other, mainly viral, infectious diseases [7], and some DNA vaccines against TB are being developed [8]. The comparative merits of RNA and DNA vaccines were reviewed in 2019 [7]. However, giant strides have now been made in the science and technology underlying the efficacy and production of ivt RNA vaccines [2]. As a consequence, the manufacture, deployment and administration of small doses on a global scale has become a commercially viable proposition for combating the COVID-19 pandemic. Can this remarkable advance be exploited for tuberculosis?

The view that adequate protection against bacterial infections such as TB will require delivery of a complexity of diverse antigens by virulence-attenuated vectors such as live BCG or attenuated *M. tuberculosis* has not been discounted; current understanding of the requirements for generating protective immunity is limited [9] and multiple forms of vaccine are being investigated [10], and must continue to be investigated. However, the attainment of potent protection with a limited number of TB antigens (subunits) remains a realistic ambition that has precedents in animal models of TB [11,12] and a range of subunit candidate vaccines are in clinical trials [9,10]. The ivt RNA approach may be ideally suited for the subunit approach. It may arguably be the safest type of vaccine known, although clearly much remains to be learned and the long-term safety remains to be established. If the key to induction of protective immunity against TB is the intensive generation of endogenous antigen within antigen-presenting cells, as may be inferred from DNA vaccine studies [11,12], then mRNA vaccines might be superior to DNA in this respect; the mRNA can be extensively engineered for enhanced *in vivo* stability and antigen production. The approach is otherwise similar to DNA vaccinology; templates for multiple antigens can be used and it avoids the complications introduced by vectors such as whole bacteria or viruses that contain components that inhibit the required immune responses. Currently, mRNA is expensive to make, but manufacturing costs will surely fall in response to demand. Furthermore, pure mRNA is inherently highly stable, for example when freeze-dried in a sealed vial, so future vaccines may not require a cold chain for delivery if adequate heat-stable adjuvanting can be accomplished [13]. Multiple antigens may be transcribed in vitro, combined and shipped as dried heat-stable doses. This advance in vaccinology will, of course, be further exploited against other emerging infectious diseases, but is there currently any other infectious agent that is likely to kill people on the scale of COVID-19 and TB? The global TB epidemic, with over a million deaths a year, surely calls for an intensive R&D effort to yield TB ivt RNA vaccines.
